# Supplementary material for: Comparative Ubiquitome Analysis Reveals Deubiquitinating Effects Induced by Wolbachia Infection in Drosophila melanogaster
Source: Int J Mol Sci. 2022 Aug 21;23(16):9459. doi: 10.3390/ijms23169459 (PMC9409319; doi:10.3390/ijms23169459)
Supplement: Supplementary file 1 [file ijms-23-09459-s001.zip › Table S2 Hatch rates.pdf]

Table S2 Hatch rates of eggs derived from cross groups with gene knockdown males

Cross group	Cross flies (male ×female)	Egg hatch rate (%)	Eggs counted	*p*-value
1	*bamGal4> w*$^-$ × Dmel T	87.30 ± 2.42	2531	
2	*bamGal4> Prosα7-hp* × Dmel T	56.43 ± 1.96	1830	2 *vs*. 1, *p*<0.001
3	*bamGal4 >Rpt4R-hp* × Dmel T	39.17 ± 3.65	1119	3 *vs*. 1, *p*<0.001
4	*bamGal4 >Rpn6 -hp* × Dmel T	0	1484	4 *vs*. 1, *p*<0.001
5	*bamGal4>Rpn7-hp* × Dmel T	0	1707	5 *vs*. 1, *p*<0.001
6	*bamGal4> Prosα7-hp* ×Dmel *w*Mel	67.45 ± 1.20	2074	6 *vs*. 2, *p*<0.01
7	*bamGal4 >Rpn6 -hp* × Dmel *w*Mel	0	1749	
8	*bamGal4 >Rpn7 -hp* ×Dmel *w*Mel	0	1311	
